# Potential Effectiveness of Registered Dietitian Nutritionists in Healthy Behavior Interventions for Managing Type 2 Diabetes in Older Adults: A Systematic Review

**DOI:** 10.3389/fnut.2021.737410

**Published:** 2022-01-24

**Authors:** Laurel Dobrow, Isabella Estrada, Nasira Burkholder-Cooley, John Miklavcic

**Affiliations:** ^1^Schmid College of Science and Technology, Chapman University, Orange, CA, United States; ^2^School of Pharmacy, Chapman University, Irvine, CA, United States

**Keywords:** hemoglobin A1c (HbA1c), blood pressure, cholesterol, self-efficacy, medical nutrition therapy (MNT)

## Abstract

**Purpose:**

A systematic review was conducted to assess how the involvement of a registered dietitian nutritionist (RDN) in healthy behavior interventions (HBIs) potentially affects outcomes in older adults with type 2 diabetes (T2D).

**Methods:**

Literature was searched for primary research published between 2016 and 2020 on HBI involving a RDN affecting outcomes in older adults with T2D. Evaluations of hemoglobin A1c (HbA1c), blood glucose, blood pressure, cholesterol, anthropometry, body composition, medication usage, healthcare cost, and self-efficacy and/or adherence to healthy behaviors outcomes were selected for inclusion. All the literature included were summarized, evaluated for certainty of evidence criteria, and assessed for bias.

**Results:**

A total of 12 studies were included for assessment. Involvement of a RDN in HBI was shown to reduce HbA1c, fasting blood glucose, low-density lipoprotein (LDL) cholesterol, and blood pressure and improve lean body mass, body mass index (BMI), and self-efficacy in populations of older adults with T2D. Compared to older adults with T2D receiving HBI involving RDNs, patients receiving usual care may incur higher healthcare costs or longer hospital stays. There was a high certainty of evidence for a RDN involvement in HBI with regard to reduction in HbA1c. There was a moderate certainty of evidence for a RDN involvement in HBI with regard to favorable changes in weight or body composition and cardiometabolic health outcomes. Statistically significant improvements in outcomes were usually sustained in follow-up after conclusion of HBI.

**Conclusion:**

RDNs may play an integral role in HBIs resulting in improved glycemic control, weight management, cardiovascular outcomes, and presumably comorbidity management. RDNs are important facilitators of diet education and nutrition assessment, which are essential in T2D management and should, therefore, be considered for routine inclusion in interprofessional teams for improved outcomes in older adults with T2D.

## Introduction

As of 2018, 26.9 million individuals in the United States have been diagnosed with diabetes with 90–95% of those patients having type 2 diabetes (T2D) (1). Poorly controlled T2D can lead to the development of various pathologies (2). Comorbidities including hypertension, obesity, dyslipidemia, and peripheral neuropathy contribute to the high societal and economic burden and decreased quality of life in T2D (3). Management of T2D typically consists of education with respect to the pathophysiology of T2D with diet and lifestyle changes that can mitigate T2D-related symptoms. Interventions emphasizing healthy behaviors in T2D that promote exercise and dietary changes can assist in weight management to positively impact the trajectory of T2D (2), lower hemoglobin A1c (HbA1c) levels, and reduce symptoms associated with comorbid diseases (4). Reduction in fat mass not only lowers individual risk of developing T2D, but also minimizes the impacts of T2D-related comorbidity such as heart disease (5). Maintaining glycemic control has the potential to reduce complications of T2D including cardiovascular events (4). While the domains of T2D management are within the scope of practice of a registered dietitian nutritionist (RDN), a summative assessment on the strength of evidence relating intervention with a RDN to specific outcomes in populations with T2D is needed. This systematic review evaluates the evidence relating healthy behavior intervention (HBI) with a RDN to outcomes in older adults with T2D.

### Demographic of Population With T2D

The risk of developing T2D increases particularly after the age of 45 years (6). In 2016, 15% of the United States population was over the age of 65 years and a 7% increase in this demographic is expected by the year 2040 (7). A similar trajectory is expected on a global scale wherein ~9% of the global population aged 65 years or older in 2019 is expected to increase to 16% by 2050 (8). By 2050, 80% of adults aged 65 years and over in the United States are expected to be experiencing two or more chronic illnesses (9), with an estimated 33% expected to develop T2D, which is a 23% increase from current population prevalence (10). An unprecedented increase in the aging population requires tailored interventions delivered by appropriate healthcare professionals to circumvent the impacts associated with increased prevalence of T2D and comorbidity.

### Healthy Behavior Intervention

Healthy behavior intervention is intended to influence individual health behaviors to improve overall health while limiting negative disease-specific outcomes (11). Nutrition counseling, exercise, and engaging in chronic disease-related education are notable elements of HBI that can positively impact health outcomes. HBI constitutes a strategy with high likelihood of positively impacting outcomes in T2D. For example, HBI including group exercise, health education, and goal setting may improve HbA1c, body mass index (BMI), and blood pressure in patients with T2D (12). HBI including a RDN may also improve self-efficacy, adherence to medications, and reduce the burden of comorbidity.

### Registered Dietitian Nutritionist Scope of Practice

Registered dietitian nutritionists utilize an evidence-based approach to prevent or delay disease development and manage acute and chronic disorders (13). Provision of medical nutrition therapy by a RDN is executed following the nutrition care process consisting of nutrition assessment, diagnosis, intervention, and monitoring and evaluation (13). RDNs are part of a multidisciplinary team and work proactively with patients to develop individualized goals and a care plan appropriate to lifestyle choices, frequency of visits, and existing medical conditions of patient. The nutrition assessment includes a nutrition-focused physical examination where body systems, oral health, muscle wasting, and appetite can be evaluated (13). Relevant tests to determine nutrition status conducted by or ordered by a RDN include blood pressure, weight, height, waist circumference, BMI, skinfold thickness, blood glucose levels, and a blood lipid panel (13). A nutrition care plan may initially involve RDN-led nutrition education to the patient that emphasizes the importance of diet prescriptions and why adherence is critical for improved health outcomes. Follow-up nutrition counseling sessions help to guide patients through diet modifications and lifestyle recommendations (13). RDNs also collaborate with case managers, physicians, nurses, pharmacists, speech pathologists, and other health professionals involved in patient care. They counsel patients on food–/nutrient–drug interactions, advise on nutrition-related plans, and are responsible for accounting for prescribed diets, medical foods, dietary supplements, and patient-centered nutrient and energy requirements (13). It is important to note that the comprehensiveness with which the scope of practice is implemented may be limited in some situations, since RDNs provide care in various settings including inpatient, outpatient, community, public, private, and in individual or group environments. RDNs are integral health professionals that provide health and wellness coaching, physical activity counseling, lifestyle advice, and health education as preventive and therapeutic care.

#### Canadian Diabetes Educator Certification Board (CDECB) and Certified Diabetes Care and Education Specialist (CDCES)

In the United States, nearly 20,000 healthcare professionals are designated CDCES by the Certification Board for Diabetes Care and Education (CBDCE). Eligibility for the designation includes diabetes education for a minimum of 1,000 h and at least 15 h of continuing education related to diabetes (14). Forty-two percent of CDCES-certified professionals are RDNs and 48% are registered nurses (RNs) (14). Similarly, the CDECB designates the Certified Diabetes Educator (CDE) credential. Providers with CDE credential must be a licensed health professional in Canada, be engaged in a diabetes training program that meets CDECB standards, and have a minimum of 800 h practicum experience (15). As of 2017, there were ~2,200 CDE providers in Canada (16).

Patients with T2D that engage with the CDE or equivalent have access to more comprehensive T2D education. In a 6-month randomized clinical trial, 70 patients receiving T2D education from the CDE had a greater HbA1c reduction (9.8 to 8.8%) compared to control (9.9–9.3%) (17). In this regard, it can be concluded that a RDN certified as a diabetes educator has the aptitude to assist a patient with T2D education and improve associated health outcomes. These professionals are largely underutilized, as it is estimated that only 25% of those diagnosed with T2D in Canada utilize these diabetes education services, with usage even lower among populations aged 65–79 years (18). Therefore, the aim of this systematic review was to determine whether the involvement of a RDN in HBI for older adults with T2D improved glycemic control, body composition, cardiometabolic outcomes, self-efficacy, medication use, and healthcare cost.

## Methods

**Figure 1:**
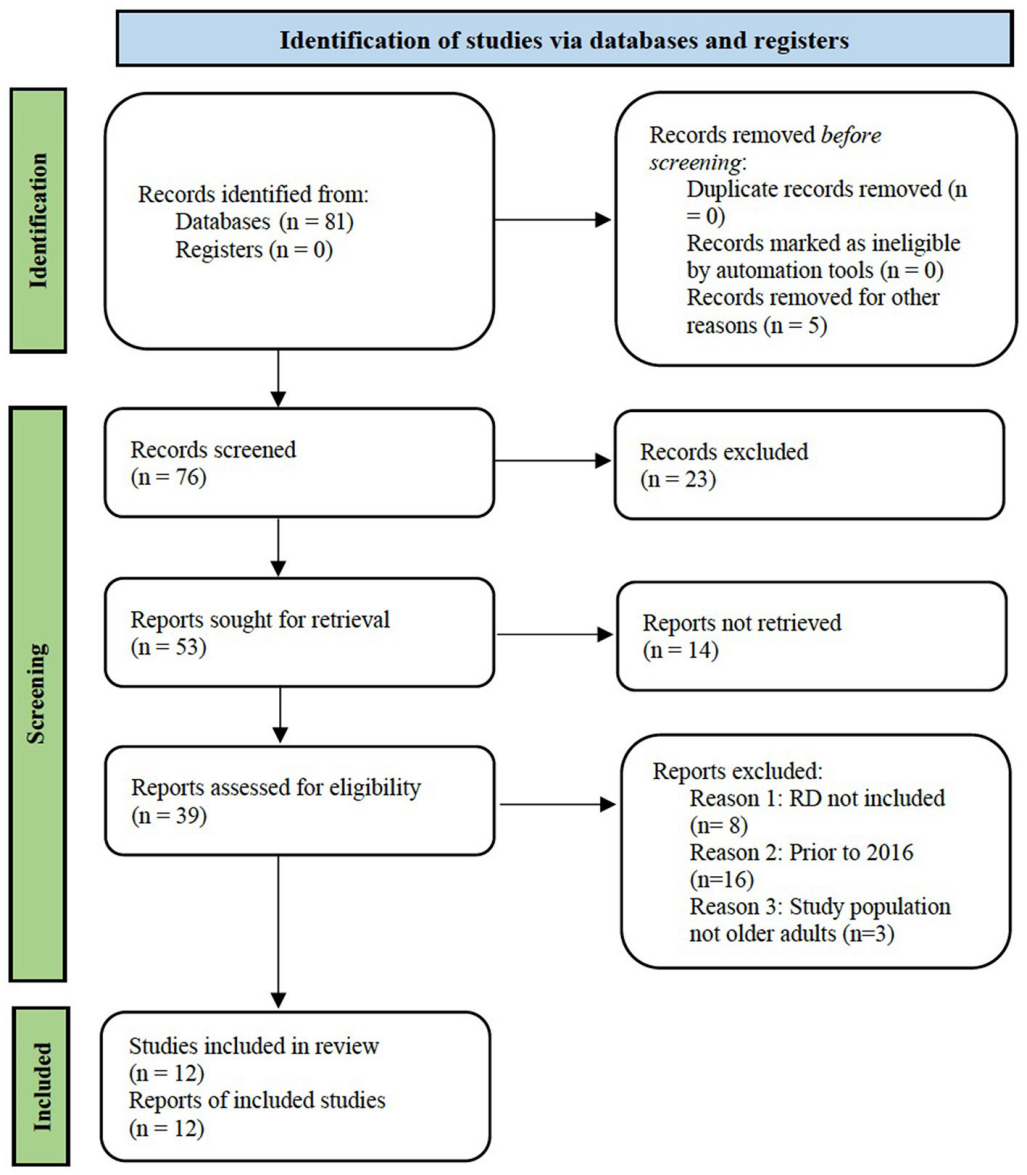
Flowchart of article identification, screening, and selection process for systematic review.

Inclusion criteria required a RDN involvement in HBI of older adults with T2D. Research studies included in this systematic review were searched and retrieved from GoogleScholar, PubMed, and Chapman University Leatherby Libraries database on or before December 10, 2020. Search terms for research published from 2016 to 2020 included: “type 2 diabetes or comorbidity,” and “older adults,” and “diet or registered dietitian or interprofessional team,” and “(healthy behavior) intervention or exercise or counseling or medical nutrition therapy (MNT).” Outcomes included HbA1c, BMI, body composition, weight, cholesterol, blood pressure, number of medications, healthcare costs, adherence to healthy behaviors, and indicators of self-efficacy (Figure 1).

Study abstracts from search results were screened. Research studies could be excluded by a single author or consensus from two of the authors' review was reached prior to selection for inclusion. Articles were excluded if studies were not interventions, did not include a credentialed RDN in the intervention, or did not have an outcome related to glycemic control, body composition, cardiometabolic outcomes, self-efficacy, medication use, or healthcare cost (Figure 1).

The systematic review followed the preferred reporting items for systematic reviews and meta-analyses (PRISMA) guidelines and included literature was assessed for the level of certainty of evidence following the grading of recommendations assessment, development and evaluation (GRADE) approach considering risk of bias, inconsistency, and indirectness (19). Variability in HBI included components of the intervention, length, frequency and a RDN contact, and range of instruments or surveys used to assess outcomes. As a result, the certainty assessment aimed to inform whether there is a potential effect of a RDN involvement in HBI on a series of outcomes, rather than on certainty of an effect estimate for a given outcome.

A high level of certainty was defined as >5 studies, with at least 50% of the studies showing statistically significant improvement for a particular outcome relative to a usual care group. A moderate level of certainty was defined as 5 or more studies with statistically significant improvement in an outcome over the intervention period for 50% of HBIs, the improvement was not assessed relative to a comparator group for at least 50% of studies, or if the total number of studies was only between 2 and 5 and at least 50% of the studies having statistical significance for a particular outcome. Evidence for all the other combinations of outcomes were deemed as low level of certainty.

## Results

The results of 12 studies meeting the criteria for inclusion of literature in the systematic review were summarized (Table 1).

**Table 1:** Summarized results of studies meeting literature search and review criteria.

**References**	**Study population**	**Sample Size**	**Groups**	**Intervention**	**Time**	**Outcomes**	**Results**	**Assessment of outcome after intervention**	**Comparison**
Sbroma et al. (20)	Diagnosed with T2D, mean HbA1c of 7.5%, average age of 59	222	Intervention (exercise physiologist, endocrinologist, sports medicine physician, psychologist, RDN, educator, nurse)	All participants engaged in a lifestyle intervention after medical examination and interview from psychologist. Intervention included periodic individualized nutrition visits and four RDN-led nutrition education sessions. Anthropometric measurements were compared at baseline, throughout, and post-intervention.	3 months	HbA1c, waist circumference, BMI, blood pressure	HbA1c changed by a mean value of −0.6 ± 1.1, waist circumference by −3.2 ± 4.7, and BMI by −0.9 ± 2.50. Statistically significant reduction in systolic and diastolic blood pressure.	24 months follow up, maintained improvements in BMI, weight, waist circumference, blood glucose, HbA1c, blood pressure.	Within group
Gilcharan et al. (21)	Diagnosed with T2D and overweight or obese BMI	320	Intervention (RDN) vs. usual care	Intervention (tDNA) group received meal counseling, exercise plan, and either counseling with motivational interviewing or conventional counseling. Usual care received dietary and exercise advice.	6 months	Eating self-efficacy measured through Weight Efficacy Lifestyle Questionnaire (WEL)	WEL scores for the usual care group was 121.9 ± 1.6 at baseline, with a −13.2 ± 2.1 difference at 12 months. The intervention group who received conventional counseling had a baseline WEL score of 134.7 ± 2.6, with an 11.6 ± 2.6 change at 12 months. The intervention group who received motivational interviewing counseling had a baseline WEL score of 129.1 ± 2.3, and a change of 28.9 ± 3.1 at 12 months.	12 months follow up, sustained WEL scores.	Intervention vs. usual care
Johansen et al. (22)	Diagnosed with T2D within the past 10 years, BMI 25–40, not using insulin	98	Intervention (RDN, endocrinologist for medication regulation, nurse) vs. usual care (nurse and endocrinologist)	Intervention group received counseling every 3 months, 30–60 min exercise sessions 5–6 times a week, dietary counseling, and a pedometer to monitor steps taken (recommendation to reach at least 10,000 steps/day). Usual care group received T2D information, lifestyle advice, and medical counseling every 3 months from nurse.	12 months	HbA1c, changes in blood glucose lowering medication, BMI	HbA1C reduced in the intervention group from 6.65 to 6.34%, 6.74 to 6.66% in the standard care group. 73.5% of individuals in the intervention group were able to lower their dosages of glucose lowering medication, compared to 26.4% in the usual care group. −2.01 change in BMI among intervention group, −0.69 usual care group (*p* = 0.001).	N/A	Intervention vs. usual care
Delahanty et al. (23)	Diagnosed with T2D, HbA1C between 6.5 and 11.5 and BMI > 25 kg/m^2^ (>23 kg/m^2^ if the participant was Asian)	208	Intervention (RDN)	All participants received dietary counseling either from an RDN in a conference call, in person with others, or referral to individual HBI.	12 months	Cost of each intervention	Individual HBI was the most cost effective ($591) followed by in person counseling ($1,380) and conference call ($1,814).	N/A	Intervention groups
Mottalib et al. (25)	Diagnosed with T2D and overweight or obese BMI. Ages 60 ± 10, not using insulin but other T2D medication ≥ 3 months	108	Intervention A vs. B vs. C (RDN)	Group A received individualized dietary counseling from RDN regarding eating plan, group B received individualized dietary counseling from RDN regarding meal planning, group C received the same intervention as B but over the phone.	16 weeks	HbA1c, BMI, waist circumference	No reduction in HbA1c for group A, but a reduction for group B (−0.66) and C (−0.61). BMI decreased for groups A, B, C by −0.43, −1.26, −1.06, respectively. Waist circumference (cm) decreased most in group B (−5.0 cm) and least in group A (−0.4 cm).	N/A	Intervention groups
Alonso-Dominguéz et al. (27)	25–70 years old with T2D	204	Intervention (nurse, smartphone app developed with help of RDN and physical activity experts) vs. usual care	Intervention group and usual care received dietary counseling. Intervention engaged in food workshop, exercise, and received smartphone application to assist in adherence to the Mediterranean diet.	3 months	Post-prandial glucose, blood pressure, waist circumference, BMI, adherence to Mediterranean diet through Mediterranean Diet Adherence Screener (MEDAS) questionnaire	Statistically insignificant reduction in post-prandial glucose, blood pressure, waist circumference, and BMI in the intervention group at 12 months follow up. At 12 months the intervention group received a 8.4 on the MEDAS, usual care received 7.1.	12 months follow up, sustained adherence to Mediterranean Diet.	Intervention vs. usual care
Agee et al. (28)	Diagnosed with T2D >6 months	224	Intervention (RDN, PCP) vs. usual care.	Intervention group received HBI from an RDN in addition to their PCP, usual care received care from their PCP.	12 months	HbA1c, systolic and diastolic blood pressure	The intervention group experienced a mean change of −0.8% HbA1c, a −8.2 mmHg in systolic blood pressure, and −4.3 mmHg in diastolic blood pressure, both statistically significant.	N/A	Intervention vs. usual care
Finn et al. (12)	Recent T2D diagnosis, >40 years of age, 2+ cardiovascular risk factors	164	Intervention (RDN, nurse, physical activity specialist, physician)	All participants were placed into the intervention group, all engaged in a community-based intervention program with a multidisciplinary team that lead group exercise, individual counseling, and health related seminars.	16 weeks	HbA1c targets, BMI, systolic and diastolic blood pressure, LDL, perceived quality of life (measured through EQ-VAS score)	BMI decreased by a mean value of 1.1 kg/m^2^, HbA1c targets were met by 75% of participants compared to 53% at baseline. Systolic blood pressure was reduced by an average of 8.8 mmHg, diastolic by 5.2 mmHg. Perceived quality of life increased by a value of 8. Statistically significant reduction in total and LDL at end of intervention and 1 year.	12 months follow up, all results maintained, physical activity targets met.	Within group
Miller and Akohoue (30)	African American women over 50 years of age with T2D	12	Intervention (previously with RDN)	Compared baseline to post-intervention results. Participants filled out dietary self-care questionnaire and were interviewed.	N/A, 2 year follow up results	HbA1c, systolic blood pressure, frequency of high-fat food consumption, spacing out carbohydrates throughout the day, BMI	Reduced HbA1C, reduced systolic blood pressure, reduced frequency in fatty-food consumption, and increased spacing of carbohydrates throughout the day. A statistically significant reduction in frequency of fruit and vegetable intake was observed. Internal factors such as motivation and external factors such as social support were the most prevalent facilitators and barriers. Baseline BMI 40.85, 2 year follow up 41.1.	24 months follow up, reduction in fruit and vegetable intake and increase in BMI. Maintained reduced HbA1c and systolic blood pressure. Reduced frequency of high fat food consumption, increasing in spacing carbohydrates throughout day.	Within group
Saleh et al. (33)	Newly diagnosed with T2D and >25 years of age	500	Intervention (RDN)	All participants received 1 h T2D education session from RDN upon enrollment and engaged in group discussions regarding T2D management.	18 months	T2D knowledge, self-care behaviors, and attitudes measured through a 4 part interviewer administered questionnaire	Total knowledge score pre intervention among male participants was 5.26 ± 2.73 and 5.62 ± 3.03 among female participants. Post-intervention total knowledge score was 9.12 ± 2.31 for male participants, 8.04 ± 2.69 for females. Total attitude scores pre intervention were 80.30 ± 6.61 for males, 79.63 ± 6.47 for females. Post-intervention total attitude scores changed to 85.98 ± 5.86 for males and 85.57 ± 6.25 for females. Pre intervention 8.3, 69.2, 25.8, and 86.7% of participants monitored blood glucose, exercise, engaged in foot care and stopped smoking, respectively, post-intervention 67.7, 85.2, 82.8, and 92.1%, respectively.	N/A	Within group
Miklavcic et al. (34)	Older adults with T2D and 2+ chronic conditions	132	Intervention (RDN, RN, program coordinator) vs. usual care	Intervention group experienced three in-home visits, participated in monthly group wellness program, monthly case conferencing, and care coordination.	6 months	Self-efficacy, self-management, and cost of healthcare	No significant differences across groups over 6-month period starting from baseline in self-efficacy, self-management, or cost of healthcare.	N/A	Intervention vs. usual care
Markle-Reid et al. (35)	Community-dwelling individuals 65 and older diagnosed with T2D and 2+ comorbidities	159	Intervention (RN, RDN, program coordinators, peer volunteer) vs. usual care	Intervention group received up to 3 in home visits from RDN and/or RN, group wellness sessions, care coordination from nurses, peer volunteers, and community partners. Usual care received support from DEC/PCN.	6 months	Self-management (SDSCA), cost, self-efficacy (self-efficacy for managing chronic disease scale)	Improvement in self-management among intervention group. At 6 months self-efficacy 8.27 ± 1.57 for intervention group, 8.05 ± 1.45 for usual care (*P* = 0.17), intervention cost neutral.	N/A	Intervention vs. usual care

### Hemoglobin A1c and Glycemic Control

In a study of patients with impaired glycemic control, a HBI including individualized nutrition visits and four RDN-led nutrition education sessions resulted in improved HbA1c within group (20). In another study of patients with T2D in Malaysia, participants were randomized to usual care or the Transcultural Diabetes Nutrition Algorithm (tDNA) HBI with meal plan, meal replacements, and at least 150 min of exercise weekly and either conventional counseling or motivational interviewing involving a RDN (21). After 12 months, participants in the tDNA group had greater decrease in HbA1c and yielded sustained results relative to the usual care group (21). Other studies have examined the role of an extensive lifestyle intervention on variations in HbA1c. In a study of individuals with T2D and a BMI between 25 and 40 kg/m^2^ who were not using insulin, participants were randomized to receive moderately intense HBI including counseling every 3 months, a more rigorous HBI consisting of 30–60 min exercise sessions 5–6 times a week, MNT from a RDN, and a pedometer to monitor steps taken with a recommendation to reach at least 10,000 steps/day (22). Those who received the extensive HBI including a RDN exhibited improved glycemic control as indicated by decreased dosage of glucose-lowering medication (22).

Another study analyzed the potential effectiveness of HBI by a RDN in a group, individually, or in a conference call (23). A within-group decrease in HbA1c occurred in each intervention arm (23). Participants with T2D engaging in a 6-month HBI individually had significantly decreased HbA1c (24). Meeting with a RDN for a structured meal plan or having a weekly phone call with a RDN was shown to significantly reduce HbA1c by 0.61% (25). Significant reductions in HbA1c were observed from baseline through the extent of the intervention and maintained at 1-year post-intervention for patients with T2D meeting with a RDN (26).

Compared to usual care, another HBI, which included a food workshop and visits with a RDN 3 and 12 months after study enrollment, showed improvement in post-prandial glucose level (27). Although this study did not show a statistically significant difference, the magnitude of change in outcome may have clinical significance (27). Alternatively, a study conducted in Altoona, Blair County, Pennsylvania found that low-to no-cost clinics offering the HBI resulted in statistically significant reductions in HbA1c 1-year post-intervention (28). Other studies examining the impact of extensive lifestyle interventions that emphasize education, exercise, and goal setting drew near identical conclusions (12). HbA1c was reduced by an average of 0.62% at the end of the intervention and was reduced by another 0.07% 1 year later (12). Regardless of the specific intervention, implementing a HBI under the guidance of a RDN has demonstrated improved glycemic control in older adults with T2D. Hence, their role in an interprofessional healthcare team is vital in the management of T2D.

### Body Composition and Weight

Measurements and assessments of fat mass and fat-free mass are important in the prognosis and monitoring of T2D. A study of patients with T2D not using insulin involved care from a clinical dietitian, lifestyle counseling every 3 months, took part in 30–60 min exercise sessions 5–6 times a week, and instruction to perform at least 10,000 steps/day (22). Compared to the usual care, the HBI group experienced a reduction in BMI, increase in lean muscle mass, and decrease in total body fat (22). Similarly, another study found significant reductions in weight for patients with T2D that completed the diabetes self-management education (DSME) program that involved a RDN (29). Meeting with a RDN for a structured meal plan or having a weekly phone call with a RDN significantly reduced body fat percentage, body weight, and waist circumference (25). When participants took part in a food workshop and regular visits with a RDN, a statistically insignificant reduction in waist circumference and BMI was shown (27). Other research showed similar results, demonstrating a statistically insignificant reduction in weight when meal planning was involved in treatment (21).

Registered dietitian nutritionists involved in HBI that included exercise and T2D education successfully impact anthropometric measures. In a study examining the impact of exercise and education, 97% of participants were overweight or obese based on BMI, with an average HbA1c of 7.35% at baseline (12). At the conclusion of intervention, BMI significantly decreased by a mean value of 1.1 in a 1-year HBI and the decrease was sustained 1 year after conclusion of the intervention (12). Nutrition education provided by a RDN within a HBI appears to improve body composition.

### Cardiometabolic Outcomes

Among individuals with poor glycemic control, HBI with a RDN can result in clinically significant improvements in blood triglyceride levels and blood pressure (20). A HBI including a food workshop and regular visits with a RDN yielded reduced systolic blood pressure, though the findings were not statistically significant (27). On the other hand, a study of low-to no-cost clinics offering a HBI resulted in statistically significant reductions in systolic blood pressure and diastolic blood pressure after 1 year of intervention (28). In another study of women with T2D, HBI including a RDN resulted in reduced systolic blood pressure relative to baseline after 2 years (30). A similar study found statistically significant reductions in systolic blood pressure, diastolic blood pressure, and low-density lipoprotein (LDL) cholesterol, which were maintained 1 year after the conclusion of the intervention (12).

Patients with T2D were randomized in a single-arm pre- and post-test HBI involving a RDN (24). After 6 months of intervention, high-density lipoprotein (HDL) cholesterol significantly increased and serum tumor necrosis factor significantly decreased (24). The production of proinflammatory proteins is linked with increased risk of developing comorbidity and minimization of these markers by intervention is paramount to abrogating the onset of additional comorbidities in addition to T2D. HBI involving a RDN may improve blood pressure, thus enhancing management of T2D and minimizing associated deleterious outcomes.

### Self-Efficacy and Adherence to Healthy Behaviors

Self-efficacy enables patient confidence in their ability to contribute to the maintenance and improvement of health. This can range from being confident in making healthy dietary choices, knowing how to exercise, and employing weight management strategies. Common attitudes of the elderly toward T2D include “diabetes is genetic, destined, and not a serious complication, let it come;” “diabetes self-care is difficult;” “I do not know what diabetes is;” and “doctors and nurses are important facilitators of self-care management” (31). Furthermore, a study conducted with the Sasak Tribe in Indonesia found improved self-efficacy over the intervention period relative to the control group measured with a self-efficacy questionnaire after attending two 60-min diet education sessions for T2D (32).

In a study of patients with T2D in Malaysia, participants were randomized to usual care or the tDNA HBI involving a RDN (21). Patient self-efficacy perceptions were measured by the Weight Efficacy Lifestyle (WEL) survey, which assessed emotions toward food, ability to control their serving sizes and food choices in various social settings, ability to resist eating when experiencing discomfort, and ability to resist eating when in a positive mood (21). After 12 months, the tDNA groups sustained improvements in “resisting eating when experiencing negative emotions, physical discomfort, and positive activities” the WEL scores compared to the usual care group (21). However, there were no significant differences for “resisting eating when food is available and when there is social pressure” across the two groups (21). The trend showed that patients that had greater decrease in weight and HbA1c levels yielded sustained results and enhanced self-efficacy. Acquiring the tools and skills needed for weight loss were shown to be potentially effective in developing self-efficacy when culturally appropriate nutrition advice is given in tandem with counseling. Similarly, it was found that a HBI with a RDN improved attitude toward T2D and self-care activities relative to baseline. Specifically, there was a statistically significant improvement in knowledge and attitudes toward T2D while participants partook in regular exercise, blood glucose monitoring, foot care, and reduced smoking frequency (33). In summary, such evidence suggests intervention with a RDN that helps to develop self-efficacy will promote management of T2D.

Other studies indicate limited improvements in self-efficacy when patients engage in an HBI involving a RDN. In a study of adults diagnosed with T2D experiencing at least two other self-reported chronic conditions, participants were randomized to a HBI with a RDN or usual care group for 6 months (34). No statistically significant differences in self-efficacy were observed possibly due to the high quality, comprehensive care in the usual group, thus limiting differences in measured outcomes (34). A cohort aged 65 years and above diagnosed with T2D and two or more comorbidities who engaged in a 6-month community-based HBI did not demonstrate significant improvement in self-efficacy relative to the control group (35). While the connection between a HBI involving a RDN and self-efficacy warrants further research, existing evidence indicating improvements in self-efficacy when engaging a RDN is notable and their involvement in an HBI is important for T2D management.

The adoption of program plans including a diet schedule, medication regimen, self-care, or physical activity has enhanced adherence when a RDN is involved in patient care. A HBI including a food workshop and regular visits with a RDN improved adherence to a Mediterranean diet 3- and 12-months post-implementation (27). In members of the Sasak Tribes of Indonesia with T2D, implementing a program with culturally appropriate dietary guidelines resulted in improved diet compliance compared to baseline, assessed by a 24-h recall (32). In another study in which a RDN was involved in the care of older adults with T2D, fatty food consumption decreased from 3.5 to 3 days per week, spacing of carbohydrate consumption throughout the day improved, but there was a reduction in fruit and vegetable intake (30). Furthermore, participation in a HBI involving group exercise sessions, education from an interdisciplinary team, and individualized counseling resulted in physical activity targets being met by 44.7% of the intervention group participants 1 year after the conclusion of intervention (12). In another cohort study of 162 older adults with T2D advised to meet the American Diabetes Association (ADA) guidelines, >10% received care from a RDN, thereby limiting potential adherence to interventions (36). A RDN involved in counseling is advantageous to improving diet and yielding lasting dietary changes that can positively impact disease biomarkers in T2D.

### Medication Use

Adhering to a multimedicine regimen can be tedious and potentially costly for the patient. Intervention with a RDN can reduce the total number of medications a patient will require (37). Patients with T2D and elevated BMI not using insulin were randomized to usual care or a HBI involving a RDN that included exercise and a pedometer (22). The HBI group had improved glycemic control after the 12-month intervention compared to baseline, with 73% of participants in this group able to reduce the dosage of their blood pressure-lowering medication (22). When compared to the intervention group, only 26% of those in the usual care group were able to reduce medication dosages, with 44% requiring an increase in medication (22). Engaging a RDN can improve medication adherence, lower the quantity of medication required by a patient, and subsequently improve health outcomes.

### Healthcare Cost

While the cost of a RDN may offset patients initially, the long-term health benefits to patients who receive intervention with a RDN are cost neutral. As of 2017, $327 billion was spent on care for patients with diabetes (38). As of 2013, patients aged 55–64 years spent ~$ 85,000 and patients aged > 65 years spent over $54,700 on T2D-related medical care per lifetime (39). Similarly, in a study analyzing healthcare savings in the HBI groups containing a RDN vs. usual care, the HBI group had an estimated cost savings of $4,241 per patient due to reduced length of hospital stay (40). Estimated yearly savings for the HBI groups including intervention and drug prescription costs were $1,660.60 per person (40). This study did not find a difference in hospital admissions or change in number of medicines; however, the reduced length of hospital stays resulted in cost reductions for patients in HBI including a RDN (40). A study conducted in Altoona, Blair County, Pennsylvania found that low-to no-cost clinics offering the HBI resulted in statistically significant reductions in HbA1c and systolic blood pressure and diastolic blood pressure 1 year post-intervention (28). However, a different study found that an intervention pertaining specifically to medication adherence was more cost-effective than other types of HBI (37). Furthermore, it was found that the interventions need to be implemented for over 2 years for improved health and cost benefits (37).

In another study, cost-effectiveness for an individual one-on-one HBI involving a RDN was found to be the most affordable option, at one-half and one-third the costs of group and telephone consult, respectively (23). While intervention by telephone incurred the highest cost, it had the highest incremental cost-effectiveness compared to the individual HBI (23). All three of these interventions were successful in showing that RDNs are cost-effective for improving various anthropometric, biochemical, and cardiovascular measures. Alternatively, other studies conducted in Canada examining the total healthcare cost to a patient associated with engaging a RDN in HBI was equivalent when compared to usual care groups (34, 35). In summary, research suggests that participating in a HBI including a RDN does not increase healthcare-associated cost in the management of T2D (35).

### Certainty of Evidence

Two prominent sources of bias were identified in interpreting the accumulation of literature for this systematic review. First, the composition of the intervention team for HBIs may have been multidisciplinary and so attributing effects specifically to a RDN may be challenging. Second, the length of intervention (mean = 7.6; range = 3–18 months) may influence whether involvement of a RDN in the HBI caused a change in a specific outcome measure. Risk of bias was considered negligible, since all the studies included within or among group statistical analyses. Inconsistency was considered negligible, since primary outcomes assessed had considerations made for covariate analyses. Indirectness was present for all the outcomes. For example, glycemic control could be assessed by HbA1c or blood glucose. Similarly, cardiometabolic outcomes included inflammatory cytokine measures, C-reactive protein, and blood cholesterol.

Certainty of evidence (Table 2) had a high level for a RDN involvement in HBI for older adults with T2D in regard to blood sugar management and reduction in HbA1c. The certainty of evidence (Table 2) had a moderate level for decrease in weight, improvement in body composition, or cardiometabolic health outcomes including LDL cholesterol and pressure. Self-efficacy and adherence to healthy behaviors had a low level for certainty of evidence (Table 2) on whether involvement of a RDN in HBI positively influenced these outcomes consistently in older adults with T2D. Finally, the analysis on certainty of evidence (Table 2) for cost and medication use was inconclusive due to the small number of studies conducted, the heterogeneity of data collection tools used, or the wide range of outcomes within each domain.

**Table 2:** Certainty of evidence assessment.

**Outcomes**	**Certainty level of evidence**	**Number of studies** **(***n***)**	**Statistically significant improvement** **(***n***)**	**In how many studies was the statistically significant improvement assessed relative to comparator group?** **(***n***)**	**In how many studies was the statistically significant outcome measured again (after intervention conclusion)?** **(***n***)**	**In how many studies was the statistically significant improvement sustained after intervention?** **(***n***)**
HbA1c and blood glucose	high	7	4	2	3	2
Body composition, weight	moderate	6	4	2	3	2
Cardiometabolic	moderate	6	4	1	4	4
Self-efficacy, adherence to healthy behaviors	low	5	2	2	2	2
Medications	inconclusive	1	0	N/A	N/A	N/A
Cost	inconclusive	3	0	N/A	N/A	N/A

## Discussion

Healthy behavior intervention involving a RDN resulted in reductions in HbA1c evidenced by statistically significant improvements in 4 out of 7 studies included (Table 2). Similarly, strong evidence supports a decrease in blood pressure, cholesterol, BMI, and weight upon engaging in a HBI involving a RDN (Table 2). There is a low level or sparsity of evidence supporting reductions in medications required or used, cost of care, and increase in HDL cholesterol (Table 2). Few studies were found on such outcomes or limited studies indicated statistically significant improvements.

### Challenges to Implementation

One study highlighted three challenges in expanding healthcare settings for patients with T2D to include a RDN (41). First, the time a diabetes educator was able to spend with a patient appeared to be limited, since educators may also fulfill other roles as nurses, pharmacologists, or clinical nutritionists (41). As such, balancing various job titles resulted in not only less interaction with patients, but also potentially lower quality information sessions and an unclear view of their role within a healthcare practice (41). Second, while diabetes educators may have extensive knowledge with respect to the pathophysiology, proper behavior interventions, and medication that may assist in T2D care, their training in behavior change from a psychological perspective may be limited. This makes recommendations, prescribed lifestyle changes, and information difficult for a provider to convey in a patient friendly manner and for a patient to implement (41). Finally, diabetes educators in this study also emphasized a low number of providers that could provide guidance to patients with T2D, which potentially creates a greater demand for care than providers available (41). In another study where RDNs were interviewed, some expressed that physicians did not refer patients to a RDN when needed due to the primary care provider (PCP) preferring to manage T2D dietary interventions on their own and perceiving a lack of value for what a RDN can offer (42).

### Future Directions

Registered dietitian nutritionists are vital in interprofessional teams to reduce burnout for involved healthcare professionals. The burnout rate of healthcare professionals can be assessed by the Maslach Burnout Inventory (MBI) (43). In a study analyzing the emotional exhaustion of healthcare professionals in an interprofessional team, it was found that there was a larger threshold until emotional exhaustion among these providers relative to clinicians not working as an interprofessional team (44). Additionally, cognitive behavioral teamwork influenced and predicted clinician-perceived safety of patient (44). This is important because the goal of the interprofessional team is to provide the comprehensive care of patient and ensure that care planning is conducive to all the aspects of their health and management of disease. RDNs in interprofessional teams describe their ability to expand their practice by providing direct nutrition care to patients, deliver health initiatives to the local community, and teach other primary healthcare professionals about nutrition (45). Another study showed that RDNs can work harmoniously in a general practice setting and was even beneficial for the other healthcare professionals. Similarly, it was concluded that collaborative care from a physician, nurse, and a RDN was potentially effective in T2D management and was associated with higher quality care for geriatric patients with T2D in the Middle East (46). This study showed the potential effectiveness of a multidisciplinary team and supports a collaborative approach for older adults with T2D.

Interprofessional teams consist of multiple healthcare professionals including a RDN, PCP, RN, pharmacist, physical or occupational therapists, or other providers that coordinate care planning to address the needs of a patient. The Diabetes Self-management Education and Support (DSMES) program consists of interprofessional healthcare teams that assist nearly one million individuals in the United States per year and provides comprehensive support for patients with T2D through accredited services, many of which are covered by Medicare™, Medicaid™, and some private insurance companies (47, 48). A controlled clinical trial was conducted where participants in the DSME program, engaged in six 1.5-h education sessions, were provided with an information handbook, given at-home activities, and were provided the opportunity to discuss information with others taking part in the intervention (49). Engagement in the DSMES program has been shown to reduce HbA1c (50).

### Limitations

Outcomes related to quality of life, mental health, depressive symptoms, or anxiety were not included due to the substantial heterogeneity in assessment tools used and are, thus, outside the scope of this systematic review. Outcomes related to mortality and burden of comorbidity are also outside the scope of this research. Studies on children or younger adults and older adults engaged in a HBI not involving a RDN were not applicable for the purpose of this systematic review.

## Conclusion

Effective HBIs focused on the management of T2D catered to the population of older adults have become increasingly important (51). Engaging with a RDN in the DSMES program or with the CDE or equivalent designation can improve health outcomes including reduced HbA1c, improved cardiometabolic parameters, and decreased fat mass. Many care needs of older adults with T2D can be met when healthcare settings and provider teams implement strategies for engagement of patient with a RDN. Successful approaches to T2D care are multifaceted and a comprehensive team of professionals implementing HBI should consider including a RDN can enhance management of the disease and related comorbidity.

## Data Availability Statement

Publicly available datasets were analyzed in this study. All data sets used are cited in the references section of this article.

## Author Contributions

JM was the lead supervisor, as well as the lead in determining methodology, investigation, formal analysis, conceptualization, writing review and editing, and resources. NB-C provided support in writing review and editing as well as support in investigation. IE and LD contributed to data curation and provided support in formal analysis. LD, IE, and JM contributed to visualization. LD was the lead in writing original draft and IE provided support. All authors contributed to the article and approved the submitted version.

## Funding

This study was funded by Chapman University.

## Conflict of Interest

The authors declare that the research was conducted in the absence of any commercial or financial relationships that could be construed as a potential conflict of interest.

## Publisher's Note

All claims expressed in this article are solely those of the authors and do not necessarily represent those of their affiliated organizations, or those of the publisher, the editors and the reviewers. Any product that may be evaluated in this article, or claim that may be made by its manufacturer, is not guaranteed or endorsed by the publisher.
